# Virus-Specific Immune Response in HBeAg-Negative Chronic Hepatitis B: Relationship with Clinical Profile and HBsAg Serum Levels

**DOI:** 10.1371/journal.pone.0065327

**Published:** 2013-06-04

**Authors:** Elisabetta Loggi, Florian K. Bihl, Carmela Cursaro, Camilla Granieri, Silvia Galli, Lucia Brodosi, Giuliano Furlini, Mauro Bernardi, Christian Brander, Pietro Andreone

**Affiliations:** 1 Department of Medical and Surgical Scinces, University of Bologna, Bologna, Italy; 2 Gastroenterology Section, Ospedale Regionale di Bellinzona e Valli, Ente Ospedaliero Cantonale (EOC), Bellinzona, Switzerland; 3 Department of Clinical and Experimental Medicine, Microbiology Section, University of Bologna, Bologna, Italy; 4 AIDS Research Institute IrsiCaixa - HIVACAT, Germans Trias i Pujol Hospital, Autonomous University Barcelona, Barcelona, Spain; 5 Institució Catalana de Recerca i Estudis Avançats (ICREA), Barcelona, Spain; Singapore Institute for Clinical Sciences, Singapore

## Abstract

**Background & Aims:**

The immune impairment characterizing chronic hepatitis B (cHBV) infection is thought to be the consequence of persistent exposure to viral antigens. However, the immune correlates of different clinical stages of cHBV and their relation with different levels of HBsAg have not been investigated. The aim of the present study was to evaluate the relationship between HBV-specific T cells response and the degree of in vivo HBV control and HBsAg serum levels in HBeAg-HBeAb+ cHBV.

**Methods:**

Peripheral blood mononuclear cells from 42 patients with different clinical profiles (treatment-suppressed, inactive carriers and active hepatitis) of cHBV, 6 patients with resolved HBV infection and 10 HBV-uninfected individuals were tested with overlapping peptides spanning the entire HBV proteome. The frequency and magnitude of HBV-specific T cell responses was assessed by IFNγ ELISPOT assay. Serum HBsAg was quantified with a chemiluminescent immunoassay.

**Results:**

The total breadth and magnitude of HBV-specific T cell responses did not differ significantly between the four groups. However, inactive carriers targeted preferentially the core region. In untreated patients, the breadth of the anti-core specific T cell response was inversely correlated with serum HBsAg concentrations as well as HBV-DNA and ALT levels and was significantly different in patients with HBsAg levels either above or below 1000 IU/mL. The same inverse association between anti-core T cell response and HBsAg levels was found in treated patients.

**Conclusions:**

Different clinical outcomes of cHBV infection are associated with the magnitude, breadth and specificity of the HBV-specific T cell response. Especially, robust anti-core T cell responses were found in the presence of reduced HBsAg serum levels, suggesting that core-specific T cell responses can mediate a protective effect on HBV control.

## Introduction

Chronic Hepatitis B virus (HBV) infection is a global health problem, with 350 million people infected worldwide, and estimated deaths due to HBV complications (cirrhosis, hepatocellular carcinoma) of more than 1 million every year [1]. Chronic HBV infection is a very multiform disease, characterized by several sero-virological profiles reflecting a complex interplay between viral antigen and humoral immune response and indicative of different host immune reactivities against the virus [1]. The antiviral immune response, mainly the T cell arm, is considered a key factor in determining the outcome of infection and it has been shown that chronic HBV is associated with the presence of dysfunctional immune responses [2]. These abnormalities develop generally early during the acute phase of infection, subverting subsequent viral control and leading to progressively more severe stages of HBV infection [2]–[5]. While the reasons for ineffective antiviral immunity are still not clear, the functional exhaustion of virus-specific T cells, manifested by a variety of intrinsic cell defects, such as the upregulation of inhibitory receptors and pro-apoptotic mediators, has been suggested as a major factor in loss of viral control [5]–[9]. These dysfunctions are believed to be a consequence of prolonged exposure to large amounts of viral antigens, as demonstrated for Hepatitis B e antigen (HBeAg) [10], [11], while the role of Hepatitis B surface antigen (HBsAg) in this dysfunction is more controversial.

HBsAg quantification is currently receiving renewed attention for its diagnostic-clinical role, in predicting response to antiviral treatment, and in identifying the individual’s infection status [12]–[15]. The determination of its circulating levels thus adds crucial additional and complementary information to HBV-DNA measurement. HBsAg is produced by several pathways, including i) translation of transcriptionally active cccDNA molecules, the intrahepatic virus reservoir acting as template for replication and ii) from the translation of viral genes transcribed from integrated HBV sequences in the host genome [16]. Furthermore, soluble HBsAg is present in the serum of HBV patients as subviral, not infectious particles, exceeding the number of virions by a factor of 10^2^–10^5^ [16]. It has been hypothesized that such as extensive “synthetic effort” by the virus could induce T cell response impairment and/or deletion, and acts as a decoy for the humoral immunity. Because the impact of HBsAg serum levels on HBV specific cellular and humoral immune responses has not been yet explored, we aimed to assess the HBV-specific T cell response in different stages of HBeAg-negative/Hepatitis B e antibodies (HBeAb) positive subjects, in order to define the relationship between HBV-specific T cells response and different degree of control, and in particular, its association with HBsAg serum levels.

## Patients and Methods

### Ethics Statement

The study, conforming to the ethical guidelines of Helsinki Declaration, was approved by the Ethical Committee of Azienda Ospedaliero-Universitaria di Bologna, Bologna (Italy), and all subjects gave written informed consent.

### Patients

A total of 42 patients with HBeAg-negative/anti-HBe-positive chronic HBsAg carriers and six subjects with resolved HBV infection as well as 10 HBV uninfected and non-vaccinated subjects were enrolled in this cross-sectional study at the Outpatient Clinics of Semeiotica Medica, Azienda Ospedaliero-Universitaria of Bologna (Italy). All patients were negative for antibodies to hepatitis C virus, delta virus and human immunodeficiency virus. Other causes of liver disease such as autoimmune, toxic or metabolic were excluded by accurate anamnesis and clinical and laboratory assessment. Demographic, clinical and serovirological features are shown in Table 1. Subjects were subdivided in 4 groups as following:

**Table 1:** Main serovirological and biochemical features of included patients.

	TreatmentControlled (n = 16)	InactiveCarriers (n = 13)	ChronicHepatitis B (n = 13)	ResolvedHBV Infection (n = 6)
Age	57,50 [52,2–66,5]	53 [47,5–70]	41 [34–54,5]	43 [39,2–61]
Sex Males (%)	14 (87,5%)	8 (61,5%)	8 (61,5%)	4 (67%)
HBV-DNA IU/mL Log_10_	<1,18	2,9 [2,6–3,1]	5,8 [3,9–6,7]	–-
HBsAg IU/mL	3554 [654,3 −5417]	164 [46,7–1750]	5928 [3394–11271]	–-
Genotype D/nonD (%)	14/2	NA	13/0	NA
ALT U/L	22,5 [18], [5]–[27,5]	21 [16]–[25]	44,5 [25–81,2]	26,5 [17–36,7]

Quantitative data are expressed as Quartiles (median, 25^th^ and 75^th^ percentile); NA: not available.

**1. Treatment Controlled (TC).** 16 patients with chronic hepatitis B (CHB) with stable viral suppression (HBV-DNA undetectable) treated with Lamivudine or Lamivudine plus Adefovir (median time on treatment: 108 months);

**2. Inactive Carriers (IC).** 13 subjects with persistent HBV-DNA ≤2000 IU/mL and alanine aminotransferase (ALT) in the normal range (<40 U/L), as defined by current guidelines [1]. In the cases where HBV-DNA and/or HBsAg levels exceeded three logs, the measurement of liver stiffness by Transient Elastography (Fibroscan®) was performed and showed in all cases absence of fibrosis (<6.5 kPa).

**3. Untreated CHB (CHB).** 13 patients with HBV-DNA >2000 IU/mL, fluctuating ALT, and histologically active disease [1];

**4. Resolved HBV infection (rHBV).** 6 subjects with sero-virological signs of previous, controlled HBV exposition (negative HBsAg and HBV-DNA and positive HBcAb and HBsAb).

A group of 10 healthy HBV-negative, unvaccinated subjects was enrolled as internal control of experimental procedures.

Both the IC and CHB patients were treatment-naive individuals. Their clinical definition was based on long-term observation, and not on a single time point evaluation, to avoid the misclassification caused by typical HBV-DNA fluctuations.

### Serovirology Markers and Quantitative HBsAg Determination

HBV serology (HBsAg, HBsAb, HBcAb, HBeAg and anti-HBe), hepatitis C (anti-HCV), hepatitis D (anti-HDV) and HIV (anti-HIV) were tested by chemiluminescent microparticle immunoassay (CMIA), using commercially available kits (ARCHITECT, Abbott Diagnostics, Wiesbaden, Germany). HBsAg was quantified by Architect HBsAg assay (Abbott Laboratories, dynamic range: 0.05–250 IU/mL); samples exceeding the value of 250 IU/mL were retested after dilution 1∶100 in kit diluent, as described [17]. HBV-DNA was quantified by real time-PCR (Abbott Diagnostics, lower limit of detection: 15 IU/mL). HBV genotype was identified (in patients with HBV-DNA >1000 IU/mL) by direct sequencing (Trugene®, Siemens, Milan, Italy).

### Synthetic Peptides

A set of 208, 18–mer, peptides overlapping by 10 residues and covering the complete protein sequence of HBV genotype D (reference strain ayw) was designed using PeptGen (http://www.hiv.lanl.gov/content/sequence/PEPTGEN/peptgen.html) and synthesized at the Peptide Synthesis Facility at Massachusetts General Hospital using Fmoc-chemistry.

### Lymphocyte Isolation and Antigen-Specific T-Cell Expansion

Peripheral blood mononuclear cells (PBMCs) were isolated from fresh heparinised blood by density gradient centrifugation, washed in phosphate buffer saline (PBS), counted and resuspended in RPMI 1640 supplemented with 10% heat inactivated Fetal Bovine Serum (FBS), 2 mM L-glutamine, 50 U/ml penicillin, 50 µg/ml streptomycin and 10 mM HEPES (all from Gibco Invitrogen, Milan, Italy). To establish in vitro expanded T-cell lines, 5–10×10^6^ PBMCs were split in 2 aliquots; one half was pulsed with peptide pools covering the complete HBV proteome sequence (4 µg/mL final concentration per peptide) for 90 minutes, washed, and added to the non-pulsed aliquot and cultured at 37°C. Recombinant interleukin−2 (50 IU/ml) was added on day 3 and twice a week thereafter. After 14 days of culture, cells were washed and starved overnight in IL-2 free medium before using them in an IFN-γ-based Enzyme-Linked Immunospot (ELISpot) Assays.

### Enzyme-Linked Immunospot Assays

ELISpot assay was performed using the short-term T-cell lines and the panel of 208 overlapping peptides pooled in 42 mixtures of a peptide matrix design such that each single peptide was part of two mixtures [18]. For the assay, 96–well plates (Multiscreen-IP; Millipore S.A.S., Vimodrone, Italy) were coated overnight at 4°C with 2 µg/ml capture mouse anti-human IFN-γ monoclonal antibody (1DIK; Mabtech, Nacka, Sweden). Plates were then washed six times with PBS-1% FBS and blocked with RPMI 1640–10% FBS. T-cell lines (7×10^4^/well) were added to the different matrix peptide pools and incubated overnight (18–20 hours) at 37°C with 5% CO_2_. Four wells were used as negative control, by incubating the cells in medium alone, and phytohemagglutinin was added at a concentration of 1.8 µg/ml to serve as a positive control. After washing with PBS, 100 µl of biotinylated anti-IFN-γ mAB 7-B6-1 (0.5 µg/ml, Mabtech) were added and plates were incubated for 1 hour at room temperature (RT). After an additional wash, the plates were incubated with a 1∶2000 dilution of streptavidin-coupled alkaline phosphatase (Streptavidin-ALP-PQ Mabtech) for 1 hour at RT in the dark. After washing the plates again, IFN-γ production was detected as dark spots after a short incubation of 10–20 minutes with nitroblue tetrazolium and 5-bromo-4-chloro-3-indolyl phosphate (BioRad, Segrate, Italy). The color reaction was stopped by washing plates with tap water and the plates were air-dried before counting using a AID ELISPOT Reader Unit (Autoimmun Diagnostika GmbH, Strassberg, Germany). Results were expressed as spot forming cells (SFC) per million input cells. Thresholds for positive responses were determined as either 5 spots per well (70 SFC/10^6^ input cells) or as the mean plus 3 standard deviations of the negative control wells, whichever was higher. After screening with matrix peptide pools, the individual targeted OLP was identified in a reconfirmation ELISpot assay carried out exactly as described above.

### Statistical Analysis

Statistical analysis was performed by non-parametric tests. Quantitative and qualitative variables were compared using the Mann-Whitney and the Fisher-exact tests, respectively. Correlations were performed using Spearman rank correlation. A p-value <0.05 was considered statistically significant. Data handling and analysis were performed with SPSS software for windows, version 17.0 (SPSS Inc., Chicago, IL, USA) and with Prism Software, version 13.

## Results

### 1. Serovirological and Biochemical Features

Median baseline levels of HBsAg were significantly higher in patients with TC and CHB than IC (p = 0.03 and p<0.001, respectively), although the HBsAg levels varied widely (from <1 to 5 logs), regardless of clinical category (Table 1). In the group of treatment naive patients (both IC and CHB patients, n = 26), HBsAg and HBV-DNA were correlated (r = 0.753, p<0.0001). ALT values reflect clinical definition, being within range in the subgroup of TC and the group of IC, with the exception of one patient, with abnormal value because of dysmetabolic disease (Table 1).

### 2. Qualitative Differences in HBV-specific Immune Response Characterize Different Clinical Profiles

**Figure 1:**
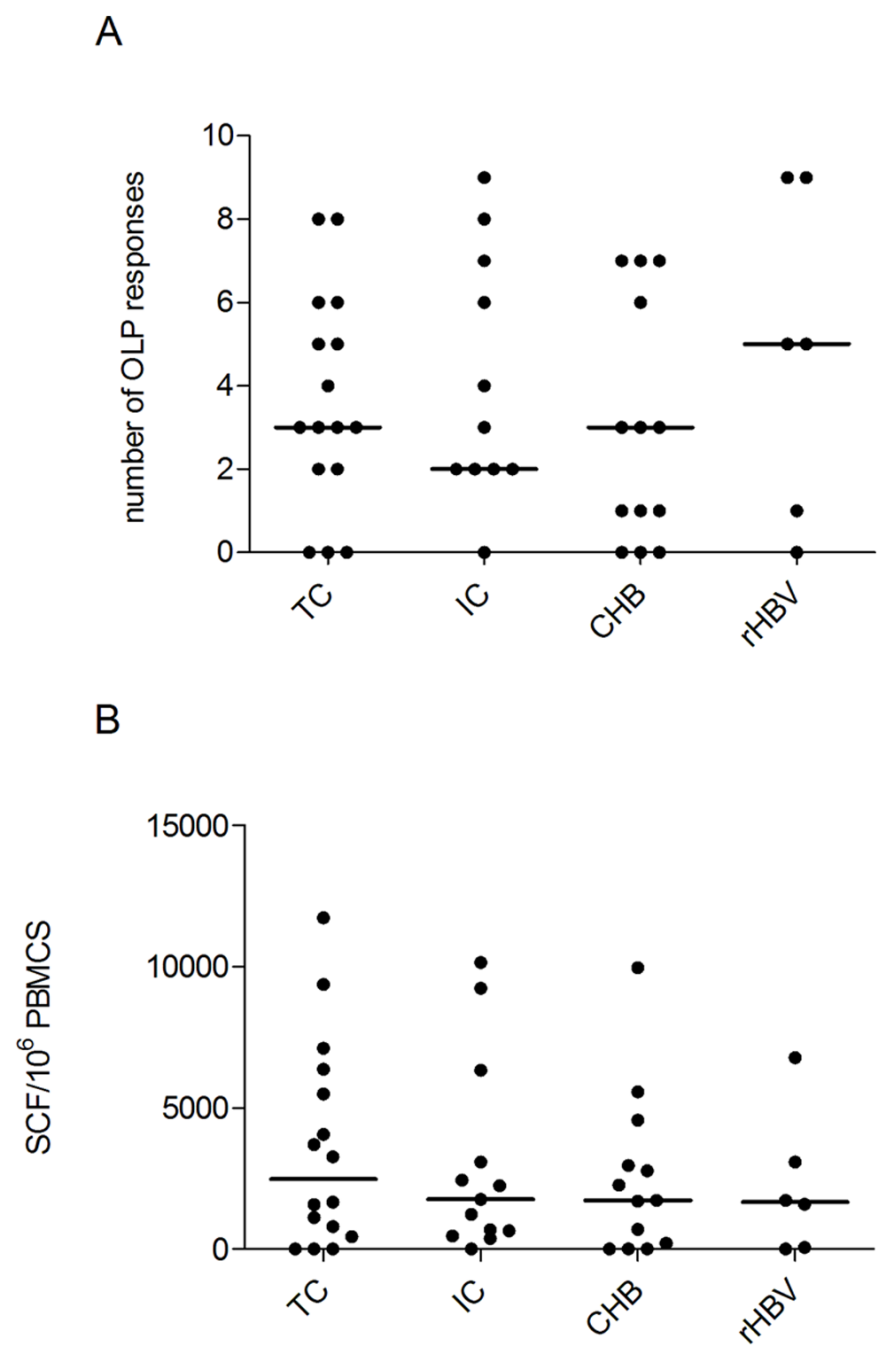
Breadth and magnitude of anti-HBV specific T cell response in the four groups of patients. A) Breadth (number of targeted peptides) and B) magnitude (number specific cells) of the anti-HBV specific T cell response in the four patients groups. Total magnitude was determined by adding magnitudes obtained for all targeted peptides by each patient. Each symbol represents an individual patient, with black horizontal bars showing the median values per patient group.

To assess if a specific immune profile can be associated to a clinical category of HBV infection, virus-specific T-cell responses in 42 anti-HBe-positive chronic HBsAg carriers with different virological profiles and in 6 patients with resolved HBV infection were tested by using ELISpot assay after in vitro T cell expansion with synthetic, overlapping peptides. A group of 10 HBV negative subjects was included as controls and did not show any reactivity to the peptides library (data not shown). Among chronically HBV infected individuals, the majority of patients showed a HBV-specific immune response, with the percentage of patients with at least one targeted sequence being marginally higher among IC (92%) compared to that of the TC and the CHB (81% and 77%, respectively, p>0.05). IFN-gamma producing T cells were detected in all but one anti-HBc-positive subjects. No significant differences in the breadth (i.e. the number of targeted peptides) nor in the total magnitude (i.e. additive magnitude of all peptide specific responses in an individual) of immune response were observed between the four groups (Figure 1), indicating that quantitative aspects of the responses do not explain the different clinical outcome between the four groups.

**Figure 2:**
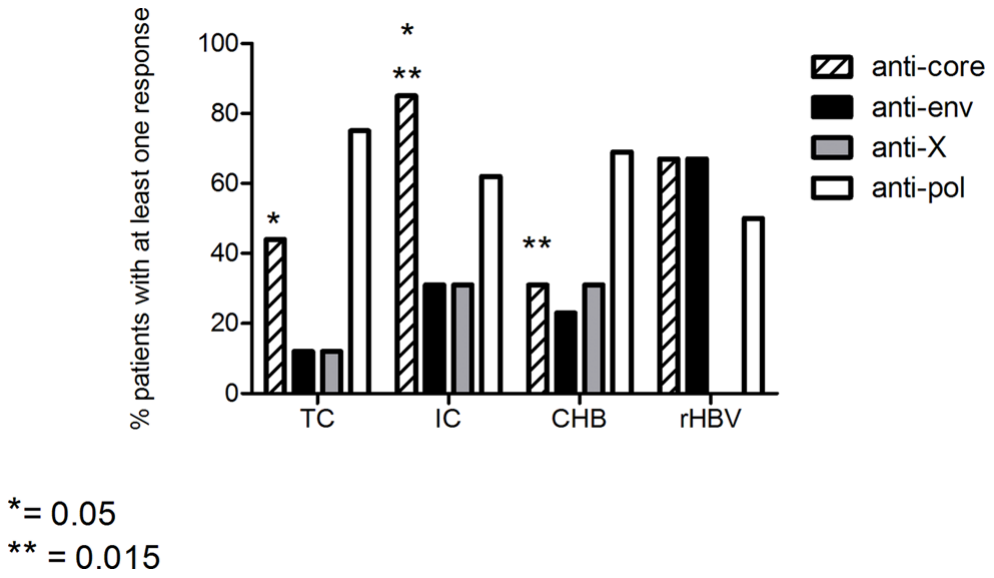
Percentage of patients with anti-HBV T cell response to the four HBV proteins in each subgroup. Percentage of patients in each clinical group with at least one response to the four different HBV proteins. Differences between percentage evaluated by Fisher’s exact test* = p value: 0.05. ** = p value: 0.015.

Overall, virus-specific T cells response was generally (75% of cases) directed towards more than one peptide and different proteins (i.e. multispecific T cell response) so that the effect of T cell specificity on HBV control could be addressed. Comparing the fine specificities of T cell responses between the four groups showed significantly different response patterns (Figure 2). Across the entire cohort, core and polymerase sequences were significantly more often targeted than X protein and envelope (54%, 67%, 21% and 27% respectively, p<0.01, data not shown). However, the IC group showed a preferential targeting of the HBV core region (85% of IC patients showed at least one anti-core T cell response), which was significantly higher than the core response rates in TC and CHB patients (p = 0.05 and p = 0.015, respectively, Figure 2). In turn, in the TC patients, the antiviral T cell reactivity was mainly directed against the polymerase protein (75% of total response), while the control group of HBV resolvers was characterized by targeting structural viral components (core and envelope), without any responses to the X protein. The response rates to the X-protein were low in the other groups as well, with response detected maximally in one third of tested individuals. Together, these data link different response patterns of the T cell response with distinct clinical outcome of HBV infection and suggest a potentially superior in vivo effectiveness of the Core and Envelope specific T cell activities.

### 3. Levels of HBsAg are Associated with Different anti-HBV Immune Profiles

**Figure 3:**
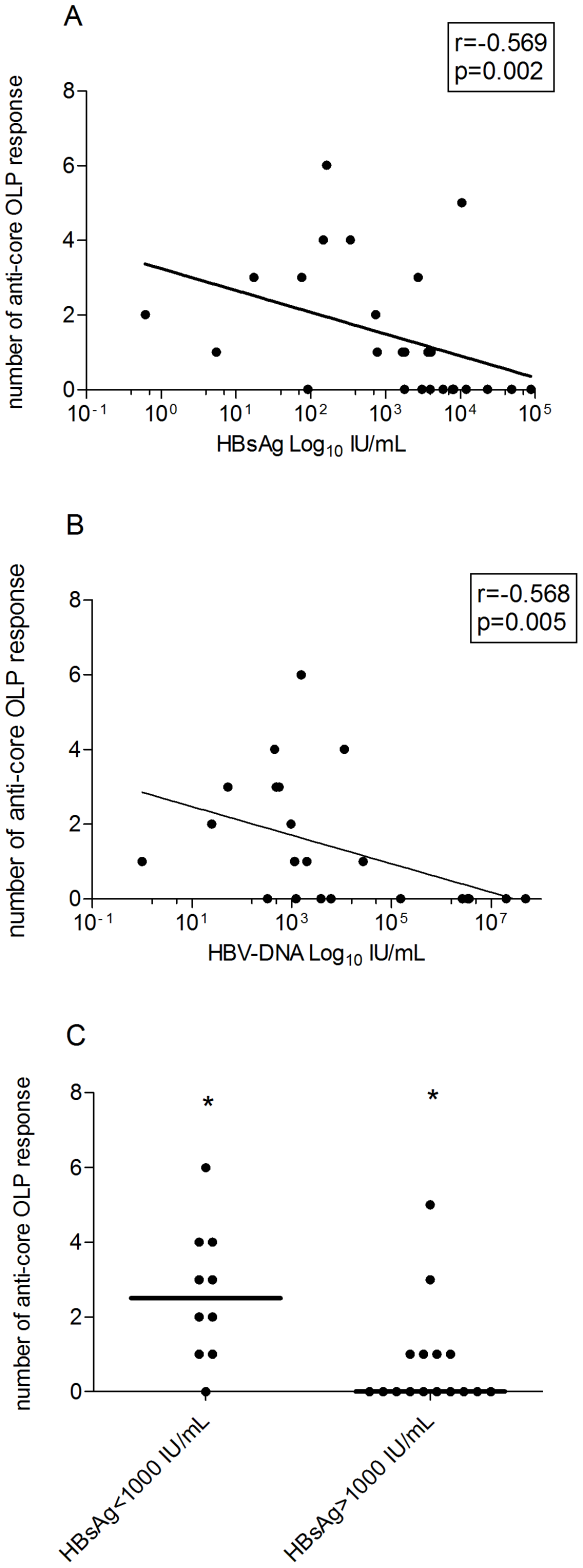
HBV-core specific T cell response and HBV serovirological markers in untreated patients (IC and CHB). A) Correlation between the breadth of the HBV core specific T cell response (number of anti-core targeted peptides) and HBsAg serum levels (IU/mL) in the 26 treatment-naive patients (IC and CHB patients). Regression line, r coefficient and p value (Spearman Ranks test) are shown. HBsAg axis in logarithmic scale. B) Correlation between the breadth of the HBV core specific T cell response and HBV-DNA levels (IU/mL) in the 26 treatment-naive patients (IC and CHB patients). Regression line, and r coefficient and p value (Spearman Ranks test) are shown. HBV-DNA axis in logarithmic scale. C) Number of anti-core responses in the 26 treatment-naive patients, stratified by level of HBsAg antigenemia (<1000 and >1000 IU/mL). Each symbol represents an individual patient with bars representing the median per group. * = p value: 0.004 (Mann Whitney).

To assess the potential relationship between antiviral immune profile and sero-virological parameters of HBV infection, the levels of serum HBsAg were compared to the frequency of protein-specific T cells response in the two treatment naive (IC and CHB) patient groups. The data show that the HBsAg serum levels were inversely correlated with the breadth of the anti-core specific T cell response (r = −0.569, p = 0.002, Figure 3A). Similarly, the HBV-DNA levels were also inversely related with the breadth of the core-specific T cell response (r = −0.568, p = 0.005, Figure 3B). In addition, serum ALT levels also positively correlated with HBsAg serum levels (r = 0.534, p = 0.006) and negatively with the anti-core response (r = −0.608, p = 0.001). The positive effect of core-specific T cell responses on HBV control was further manifested when the untreated patients in the IC and the CHB groups were stratified based on a HBsAg cut-off 1000 IU/mL, showing a significantly broader anti-core T cell response in patients with levels of HBsAg<1000 IU/mL than patients with HBsAg>1000 (median frequency 2,5 vs 0, respectively, p = 0.004, Figure 3C). Similarly, among the 15 individuals with core-specific T cell responses, the IC group was over-represented compared to the CHB (n = 11 out of 13 vs 4 out of 13, p = 0.001, Table 2).

**Table 2:** HBsAg serum levels and HBV-DNA levels in patients with or without anti-core response.

	Clinical group	HBsAg IU/mL	HBV-DNA IU/mL Log_10_
Anti-core response+(N = 15)	−11 IC^1^; 4 CHB	744^2^, [76–2742]	5,805^3^, [3], [5]–[6], [7]
Anticore-response - (N = 11)	−2 IC^1^; −9 CHB	7889^2^, [3079–23210]	3,025^3^, [2,7–3,3]

p values: 1 = 0.01; 2 = 0.002; 3 = 0.018. Quantitative data expressed as Quartiles (median, 25^th^ and 75^th^ percentile).

Overall, the data indicate that the anti-HBc response is associated with superior in vivo control of HBV infection. Interestingly, the most frequently targeted region in the core protein, which spanned the sequence LCWGELMTLATWVGVNL (position 60−76) was targeted in 6 out of 15 patients (40%) and contains an immunodominant CD4 T cell epitope often recognized during acute hepatitis [19].

This sequence was more often targeted than the sequence including the well described CD8 epitope core 18−27, previously found to quantitatively dominate the CD8 T-cell response [2], [4].

### 4. HBsAg Levels Correlate with HBV-specific T Cell Response in Treated Patients

**Figure 4:**
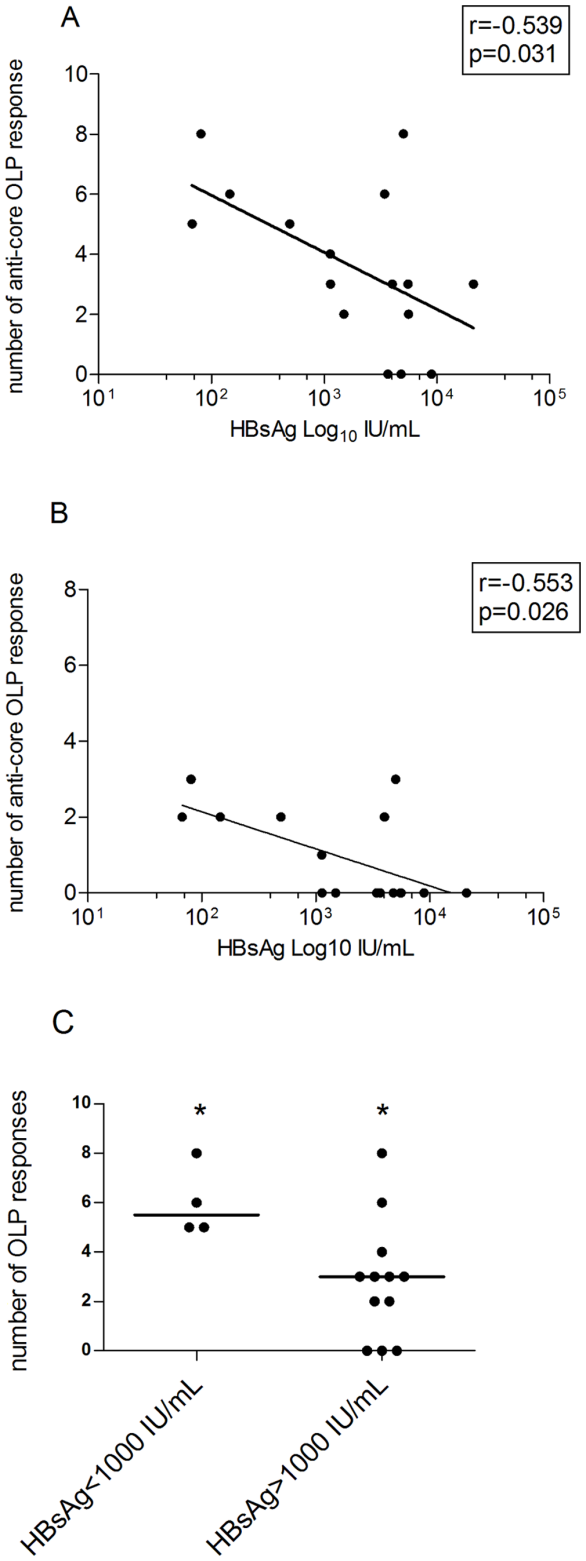
HBV specific T cell response and HBV serovirological markers in treated patients. A) Correlation between the breadth of the HBV specific T cell response (number of targeted peptides) and HBsAg serum levels (IU/mL) in the 16 treatment-suppressed patients. B) Correlation between breadth of the HBV specific T cell response and HBV-DNA serum levels (IU/mL). For A) and B), Regression line, r coefficient and p value (Spearman Ranks test) are shown. HBV-DNA axis in logarithmic scale. C) Number of responses in the 16 treated patients, stratified by level of HBsAg antigenemia (<1000 and >1000 IU/mL). Each symbol represents an individual patient with bars representing the median per group. * = p value: 0.03 (Mann Whitney).

In order to assess the impact of antiviral treatment on cellular host immunity to HBV, a group of long-term treated, virally suppressed patients was also evaluated. The duration of treatment was 108 months in median (range 31−132 months). As in the untreated population, HBsAg serum levels were inversely correlated with the overall breadth of virus-specific T responses (r = −0539, p = 0.031, Figure 4A) and, in particular, with the anti-core specific T cell response (r = −0.553, p = 0.026, Figure 4B), although the major contribution to the antiviral reactivity in this group was conferred by anti-polymerase response (Figure 2). Furthermore, dividing the patients on the basis of the above HBsAg cut-off (1000 IU/mL), both the overall breadth of response as well as the anti-core immune response alone were significantly higher in patients with lower antigenemia (median frequency 5,5 vs 3, respectively, p = 0.03, Figure 4C and data not shown). No difference in total or core-specific responses were noted when the treated individuals were stratified for the duration of treatment (<100> months). These data indicate a surprisingly robust maintenance of HBV-specific responses for extended period of time after long-term viral suppression.

## Discussion

In chronic infections, severe immune impairment, such as overexpression of inhibitory co-receptors, altered development and maintenance of memory cells, and modification in cytokine production have been described [20]. Chronic HBV infection is no exception to this as a variety of intrinsic cell defects, such as the upregulation of inhibitory PD-1 pathway and of pro-apoptotic mediators have been reported [5]–[8]. The peculiarities of HBV infection likely responsible for immune impairment are the unique features of the intrahepatic milieu, where several cell types are able to produce immunosuppressive cytokines (IL-10, TGF-β), and where persistent inflammation causes the depletion of some essential nutrients [2]. In addition, the general inefficiency of hepatocytes as antigen-presenting cells, that require higher concentrations of antigen to prime or maintain an adequate virus-specific T cells may further limit effective viral control [21]. Most importantly however, the host immune system is exposed to large amount of viral antigens, in particular of HBsAg, produced in large excess over infectious virions [2].

Several recent reports suggest that HBsAg quantification represents a very useful tool in the clinical management of chronic HBV, being able to predict the response to antiviral therapy as well as to help in optimizing the clinical classification of patients [12]–[14]. This aspect is particularly relevant in the HBeAg negative/HBeAb positive patients, where the continuous fluctuations of HBV-DNA make the disease definition often difficult. In comparison to HBV-DNA, the HBsAg levels are significantly more stable over time [15]. Although the virological correlates of HBsAg levels remain controversial, as some reports found them correlated with cccDNA [22], [23], while more recent ones did not [24], [25], variable HBsAg amounts have been postulated to reflect different degree of immune control. However, the precise relationship between HBsAg serum levels and the cellular antiviral immune response has not been investigated.

Our data identify a strong inverse association between T cells response and sero-virological parameters of HBV infection, and confirm previous observations indicating that the strength of the immune response may be severely impaired in the presence of active viral replication [4], [5].

The present results expand earlier reports and shows inverse correlation between HBsAg serum levels and anti-HBV T cell response in both, treatment-controlled and treatment-naive patients. Although the experimental set up employed here can not discriminate between CD4 and CD8 T cell responses, the study shows that the majority of patients have a HBV-specific T cell reactivity and identifies specific response patterns associated with different serovirological profiles. In the treatment-naive patients, these response patterns suggest that anti-core specific T cell response may be the hallmark of relative protection from progressive HBV disease, as it is significantly more frequent in the IC patients, and inversely correlated with HBsAg, HBV-DNA and ALT levels. This association is particularly pronounced for HBsAg, since the frequency of T cell anti-core response is significantly different in patients with HBsAg above or below 1000 IU/mL. Interestingly, this threshold has been recently proposed as a useful clinical parameter to discriminate between different clinical profiles of HBV infection [15].

Previous reports have suggested that the anti-core response is effective in the control of HBV, being detectable in patients with self-limited acute hepatitis, as well as in the phase preceding the HBeAg seroconversion [26], [27]. However, the majority of these data have been generated in individuals tested during acute phases of HBV infection, and using recombinant proteins and/or a limited number of HLA-restricted peptides. Our data, based on a comprehensive testing of all viral proteins in patient groups of variable clinical status, confirm these findings. They also show that the envelope region was rarely targeted in the chronic patients, while a robust anti-envelope response was detected only in patients with controlled infection, and which were positive for anti-S antibodies. Our results also demonstrate that the so called “inactive carrier status” is not associated with a lack of T cell responses to the virus; rather the data indicate that this favourable non-progressive status is conferred by the activity of T cells with determined specificity and which probably acts through non-cytolytic mechanisms or in a highly selective manner on virally infected cells only with little bystander damage. It is important to note that spontaneous as well as treatment-induced control of infection is associated with different immune profiles. In fact, in patients treated with nucleoside/nucleotide analogues, the predominant response was directed against the polymerase protein, although even in this group, there is an inverse association between the levels of HBsAg and T cell specific immune response, as well as with the anti-core immune response. Certainly this observation is limited by the lack of a longitudinal evaluation which prevents us to discriminate the cause and the consequence of this association, and further studies on larger samples, and with additional details on features of HBV-specific T cells (CD4/CD8) are needed. In addition, the interpretation of the present data is complicated by the reported presence of preS/S mutants, affecting the HBsAg levels in both naives and treatment-resistant patients [28], [29]. This issue, and in particular the emergence of variants as result of immune pressure needs to be further elucidated.

Despite these limitations, the present data suggest that the anti-HBV immune response in individuals with different clinical profiles of HBV infection is not quantitatively, but qualitatively different, and that core-specific T cell response may mediate particularly effective in vivo control. As such, the results also show that the common notion of universally weak T cell response in chronic hepatitis is incorrect and that specific response patterns can be associated with particular clinical profiles. Finally, the results may also help guide the development of an immunotherapeutic approach for chronic HBV, which has currently been attracting much attention [6,30., and which could possibly be optimized by directing the host T cell response to most effective targets.
